# Chronic myeloid leukemia-derived exosomes promote tumor growth through an autocrine mechanism

**DOI:** 10.1186/s12964-015-0086-x

**Published:** 2015-02-03

**Authors:** Stefania Raimondo, Laura Saieva, Chiara Corrado, Simona Fontana, Anna Flugy, Aroldo Rizzo, Giacomo De Leo, Riccardo Alessandro

**Affiliations:** Dipartimento di Biopatologia e Biotecnologie Mediche e Forensi, Università degli studi di Palermo, sezione di Biologia e Genetica, Via Divisi 83, 90100 Palermo, Italy; Azienda Ospedaliera Ospedali Riuniti Villa Sofia- Cervello, Anatomia Patologica, Palermo, Italy

**Keywords:** Exosomes, Chronic myeloid leukemia, Autocrine mechanisms, Anti-apoptotic pathways, TGF-β1

## Abstract

**Background:**

Chronic myeloid leukemia (CML) is a clonal hematopoietic stem cell disorder in which leukemic cells display a reciprocal t(9:22) chromosomal translocation that results in the formation of the chimeric BCR-ABL oncoprotein, with a constitutive tyrosine kinase activity. Consequently, BCR-ABL causes increased proliferation, inhibition of apoptosis, and altered adhesion of leukemic blasts to the bone marrow (BM) microenvironment. It has been well documented that cancer cells can generate their own signals in order to sustain their growth and survival, and recent studies have revealed the role of cancer-derived exosomes in activating signal transduction pathways involved in cancer cell proliferation. Exosomes are small vesicles of 40–100 nm in diameter that are initially formed within the endosomal compartment, and are secreted when a multivesicular body (MVB) fuses with the plasma membrane. These vesicles are released by many cell types including cancer cells, and are considered messengers in intercellular communication. We have previously shown that CML cells released exosomes able to affect the tumor microenvironment.

**Results:**

CML cells, exposed up to one week, to exosomes showed a dose-dependent increased proliferation compared with controls. Moreover, exosome treatment promotes the formation of LAMA84 colonies in methylcellulose. In a CML xenograft model, treatment of mice with exosomes caused a greater increase in tumor size compared with controls (PBS-treated mice). Real time PCR and Western Blot analysis showed, in both *in vitro* and *in vivo* samples, an increase in mRNA and protein levels of anti-apoptotic molecules, such as BCL-w, BCL-xl, and survivin, and a reduction of the pro-apoptotic molecules BAD, BAX and PUMA. We also found that TGF- β1 was enriched in CML-exosomes. Our investigations showed that exosome-stimulated proliferation of leukemia cells, as well as the exosome-mediated activation of an anti-apoptotic phenotype, can be inhibited by blocking TGF-β1 signaling.

**Conclusions:**

CML-derived exosomes promote, through an autocrine mechanism, the proliferation and survival of tumor cells, both *in vitro* and *in vivo*, by activating anti-apoptotic pathways. We propose that this mechanism is activated by a ligand-receptor interaction between TGF-β1, found in CML-derived exosomes, and the TGF- β1 receptor in CML cells.

**Electronic supplementary material:**

The online version of this article (doi:10.1186/s12964-015-0086-x) contains supplementary material, which is available to authorized users.

## Background

Chronic myeloid leukemia (CML) is a clonal hematopoietic stem cell disorder in which leukemic cells display a reciprocal t(9:22) chromosomal translocation that results in the formation of the chimeric BCR-ABL oncoprotein, with a constitutive tyrosine kinase activity [1,2]. Imatinib mesylate (IM) is a selective and well-tolerated inhibitor of the BCR-ABL tyrosine kinase, and has significantly improved the prognosis of patients with chronic phase CML. Despite this remarkable progress, a major problem associated with the administration of imatinib is acquired resistance [3]. Therefore, there is an urgent need for new anticancer agents and combinations that could improve responses and survival rates for CML. Recently, a considerable interest in the cancer field has focused on the role of the microenvironment in regulating the growth, survival, and drug-resistance of leukemic cells [4]. A variety of cytokines, growth factors, adhesion molecules, and extracellular matrix proteins are secreted by both tumor and non-tumor cells, mediating cell-to-cell communication within the tumor microenvironment, and providing a suitable niche for cancer cell growth and survival. Recently, exosomes have been considered as new vehicles of these molecules into the tumor microenvironment and, as a result, data is beginning to accumulate on their role as new actors in the crosstalk between cancer and normal cells in the tumor microenvironment. Exosomes are small vesicles of 40–100 nm in diameter that are initially formed within the endosomal compartment, and are secreted when a multivesicular body (MVB) fuses with the plasma membrane [5]. We and other groups have recently shown that cancer-derived exosomes modulate the crosstalk between leukemia cells and the bone marrow microenvironment [6,7]. In particular, we reported that CML cells release exosomes, and that the addition of these vesicles to vascular endothelial cells, as well as to bone marrow stromal cells, affects *in vitro* and *in vivo* tumor progression [6,8,9]. While increasing evidence is accumulating on the role of exosomes in mediating paracrine interplays within the tumor microenvironment, little is known about their role in affecting the growth and survival of the releasing cells [10]. A regulated orchestration of both survival and death pathways is essential for a variety of normal biological processes, and disruptions of this balance often lead to a cancer phenotype [11]. Therefore, a better understanding of the molecular events that allow tumors to evade apoptotic death should enable a more rational approach to anticancer drug design and therapy. To the best of our knowledge, little is known about the role of exosomes in influencing the balance between pro- and anti- apoptotic pathways. A key regulator of the balance between life and death is the transforming growth factor β1 (TGF- β1), a multifunctional cytokine that regulates growth, differentiation, apoptosis and migration of various types of cells, including cancer cells [12]. Various studies suggest that TGF-β1 activates PI3K/Akt/NF-kB/MMP9 signaling pathways in Philadelphia chromosome-positive CML hemangioblasts [13-15]. Moreover, evidence suggests that tumor exosomes express membrane-associated TGF-β1 [16,17]. Here we show that LAMA84-derived exosomes are able to promote, through an autocrine mechanism, the proliferation and survival of tumor cells, both *in vitro* and *in vivo*, by the activation of an anti-apoptotic pathway regulated by exosome-associated TGF- β1.

## Results

### LAMA84-derived exosomes characterization

Exosomes from LAMA84 culture supernatant were isolated and described by our group in 2012 [9]. Taverna et al. by using scanning electron microscopy showed that CML vesicles consisted of a homogenous membrane particles population with an average diameter of 70 nm ± 10. They also showed that exosomes were positive for CD63 and Hsc70.

Here, we further characterized LAMA84-derived exosomes for the presence of other molecular markers, CD81, Alix and Tsg101. Importantly, exosomes are negative for Calnexin, an endoplasmic reticulum marker (Additional file 1: Figure S1).

### LAMA84-derived exosomes promote *in vitro* and *in vivo* tumor growth

**Figure 1:**
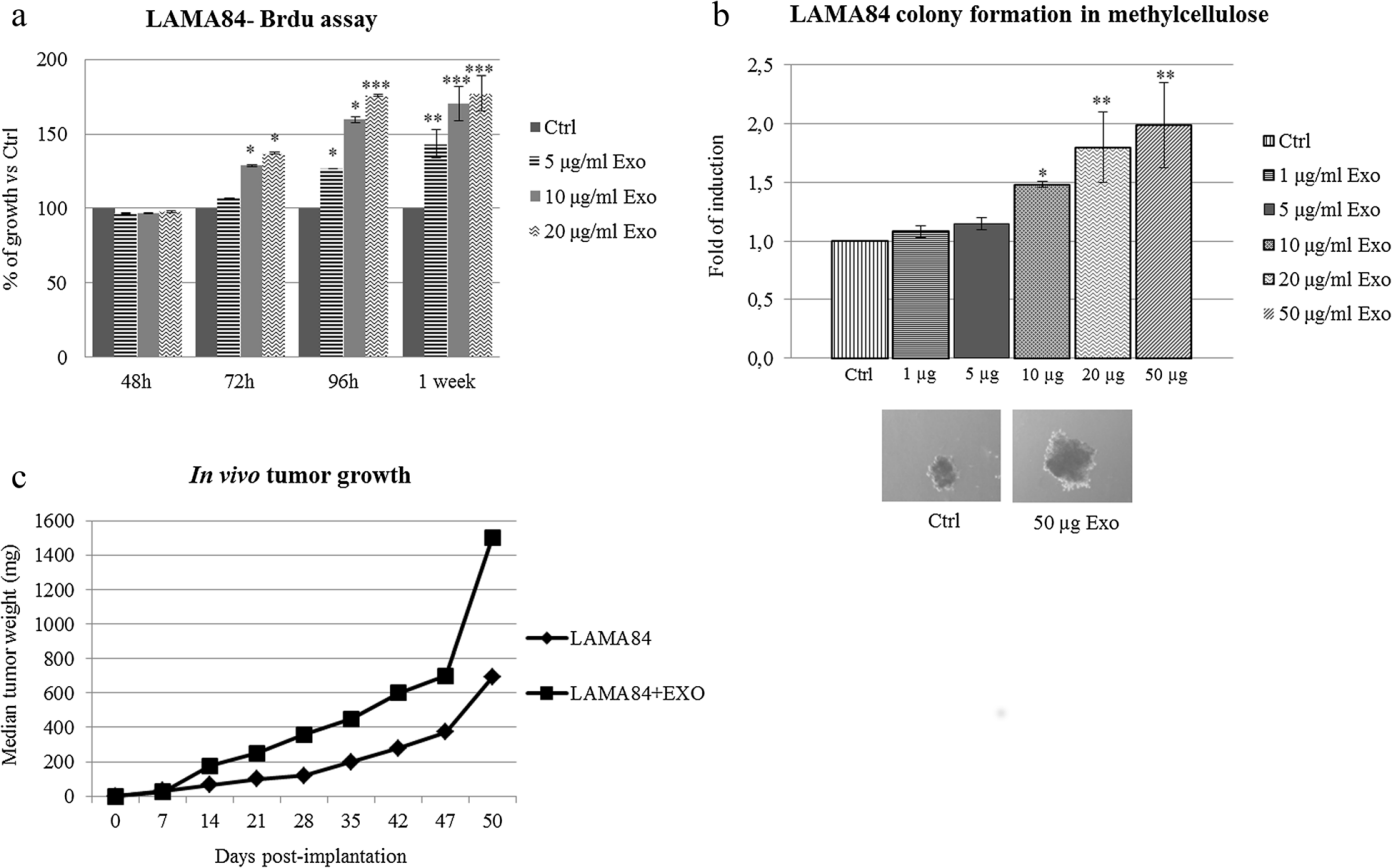
**CML-Derived Exosomes Promote** ***in Vitro*** **and** ***in Vivo*** **Tumor Growth.** Cell growth was evaluated by BrdU assay **(a)**. For the experiment LAMA84 cells were incubated for 48, 72, or 96 hours, or 1 week, with increasing doses of LAMA84-exosomes (5–10–20 μg/ml). The values were plotted as a percentage of the control (untreated cells); each point represents the mean ± SD for three independent experiments. **(b)** The addition of 1, 5, 10, 20 or 50 μg/ml of LAMA84 exosomes increased the area of CML colonies in methylcellulose compared with controls. Asterisks indicate a statistically significant values in comparison to control (Ctrl) (*p ≤ 0.05; **p ≤ 0.01; *** p ≤ 0.001). **(c)** LAMA84 cells were injected subcutaneously in NOD/SCID mice, and mice were treated as described in Material and Methods. Comparison of the median tumor weight was used as an index of the proliferative efficacy of CML-derived exosomes.

In order to assess the ability of LAMA84-derived exosomes to promote tumor growth, FBS-deprived LAMA84 cells were treated for 48, 72, or 96 hours, or 1 week, with escalating doses of exosomes (5, 10 or 20 μg/ml). The BrdU proliferation assay confirmed that at 72 hours of exosome treatment there is an increase in the proliferation rate of CML cells compared with untreated cells (Figure 1a). Similar data were obtained using MTT assay (data not shown). To better evaluate the ability of CML-derived exosomes to promote *in vitro* tumor growth, we performed a colony formation assay in methylcellulose. As shown in Figure 1b, LAMA84 cells treated with 1, 5, 10, 20 or 50 μg/ml of LAMA84-exosomes are able to form colonies in methylcellulose with a greater area than control cells.

The ability of CML exosomes to promote tumor growth was also tested in an *in vivo* tumor xenograft model. LAMA84 cells were inoculated subcutaneously in NOD/SCID mice, and the mice were subsequently treated locally twice a week with vehicle (PBS) or LAMA84-derived exosomes. After 50 days, the mice were sacrificed, and the tumors removed to measure tumor weight. Tumor weight curve in Figure 1c shows that exosome-treated mice developed larger tumors compared with control mice.

### LAMA84-derived exosomes affect the balance between pro- and anti-apoptotic molecules

**Figure 2:**
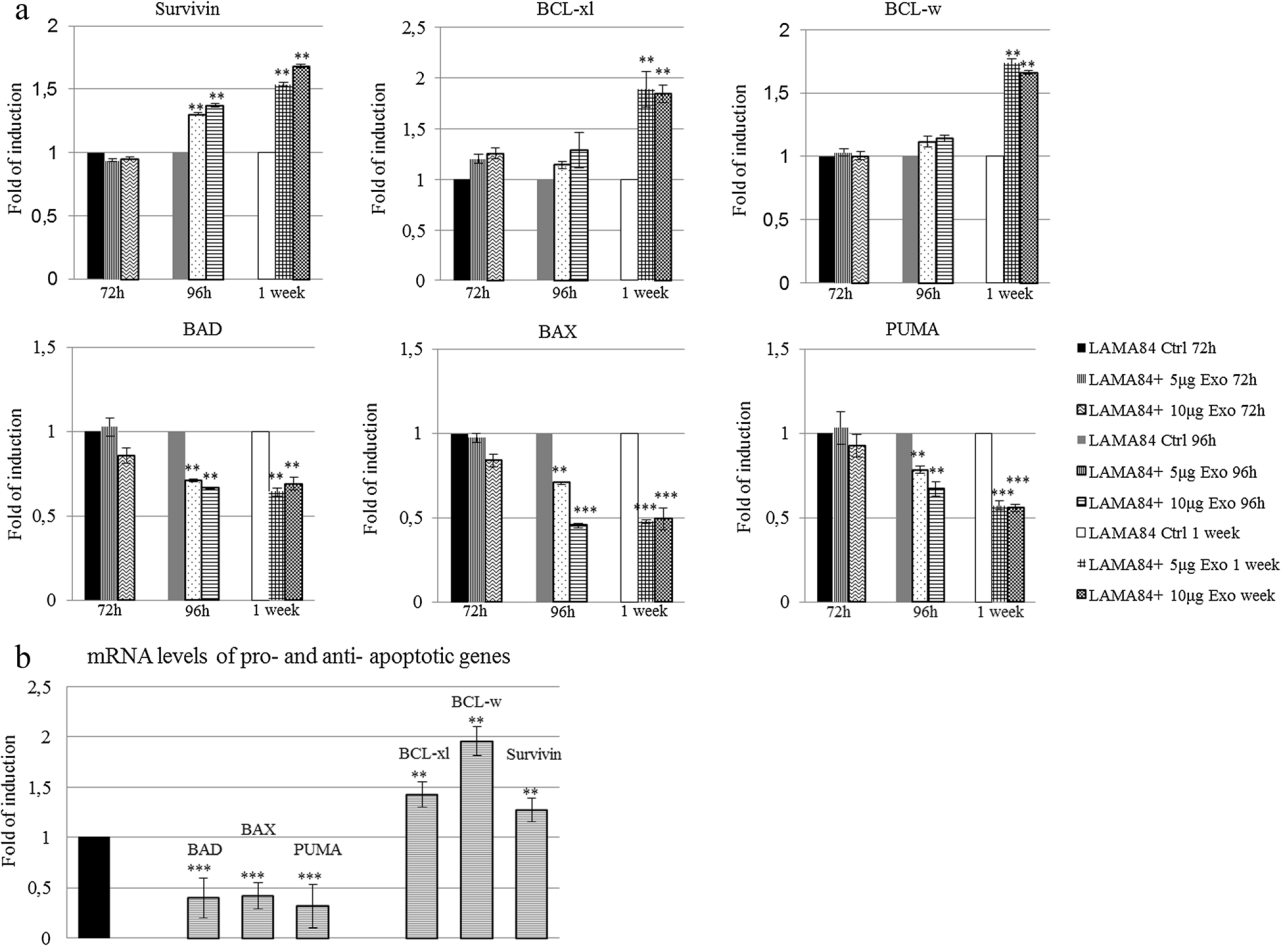
**Anti-Apoptotic and Pro-Survival Effect of CML-Derived Exosomes on Gene Expression. (a)** Real-time PCR analysis shows that treatment of LAMA84 with LAMA84 exosomes up to 1 week increases the mRNA expression of the anti-apoptotic genes survivin, BCL-xl, and BCL-w, while it decreases the mRNA levels of the pro-apoptotic genes BAD, BAX and PUMA. **(b)** Similar effects were observed in samples from mice treated with exosomes compared with control mice, treated with PBS (black bar). Asteriks indicate a significant difference in comparison to control (Ctrl) (**p ≤ 0.01; *** p ≤ 0.001).

**Figure 3:**
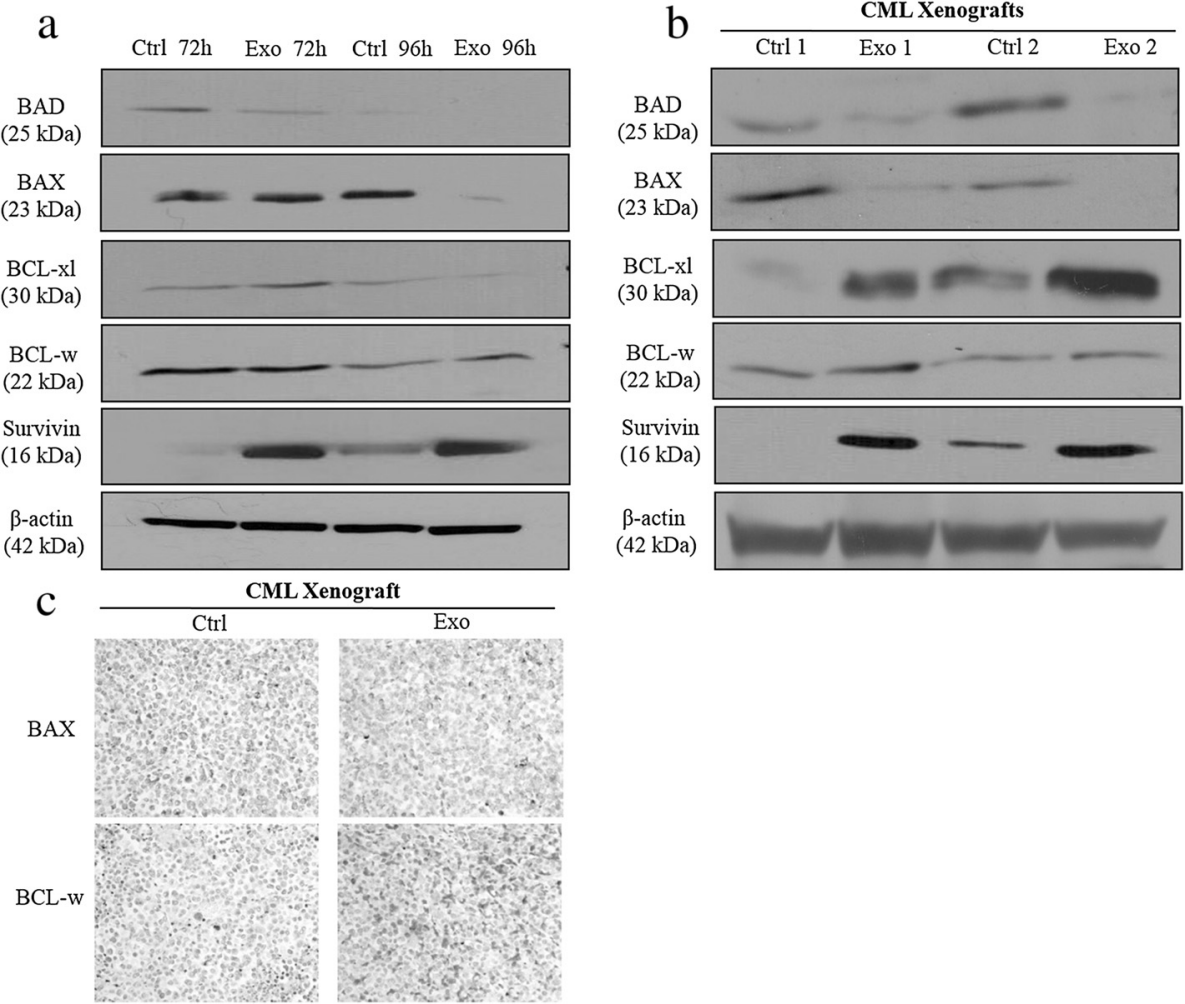
**Anti-Apoptotic and Pro-Survival Effect of CML-Derived Exosomes on Protein Expression. (a)** Western blot analysis shows that treatment of LAMA84 with 10 μg/ml of LAMA84 exosomes for 72 or 96 hours increases the expression of the anti-apoptotic molecules BCL-xl, BCL-w, and survivin, while it decreases the expression of the pro-apoptotic molecules BAD and BAX. **(b)** Protein levels of the same molecules were analyzed in the tumor biopsies of mice treated with exosomes (Exo 1 and Exo 2) and control mice treated with PBS (Ctrl 1 and Ctrl 2). Blots were stripped and subsequently reprobed with an antibody against β -actin to ensure equal loading. **(c)** Representative photomicrographs showing 5-μm-thick paraffin-embedded sections of tumor biopsy specimens obtained from control mice (Ctrl) and exosome-treated mice (Exo) stained for BAX and BCL-w.

To evaluate the mechanism by which CML-exosomes are able to sustain tumor growth, we tested the expression of different molecules involved in the apoptotic machinery. As shown in Figure 2a, LAMA84 treated for 72, 96 hours, or 1 week, with 5 or 10 μg/ml of LAMA84-derived exosomes showed a reduction of the pro-apoptotic genes BAD, BAX and PUMA and an increase in mRNA levels of the anti-apoptotic genes survivin, BCL-xl, and BCL-w. Real-time PCR analysis of the mRNAs isolated from *in vivo* xenograft tumors confirmed the data obtained *in vitro* (Figure 2b), thus suggesting that tumor exosomes are able to affect the balance between the pro- and anti-apoptotic molecules. These results were confirmed with Western blot analysis. Specifically, Figure 3a shows that in exosome-treated LAMA84 cells there is the reduction of BAD and BAX proteins, as well as an increase in the protein levels of BCL-xl, BCL-w, and survivin compared with untreated cells after 72 or 96 hours of exosome treatment (10 μg/ml). Western blot analysis of *in vivo* samples confirmed the *in vitro* data (Figure 3b). The decreased expression of BAX, as well as the increased expression of BCL-w, was also confirmed by immunohistochemical analysis on tumor biopsies (Figure 3c).

### CML-derived exosomes promote the activation of ERK, Akt and NF-kB in LAMA84 treated cells

**Figure 4:**
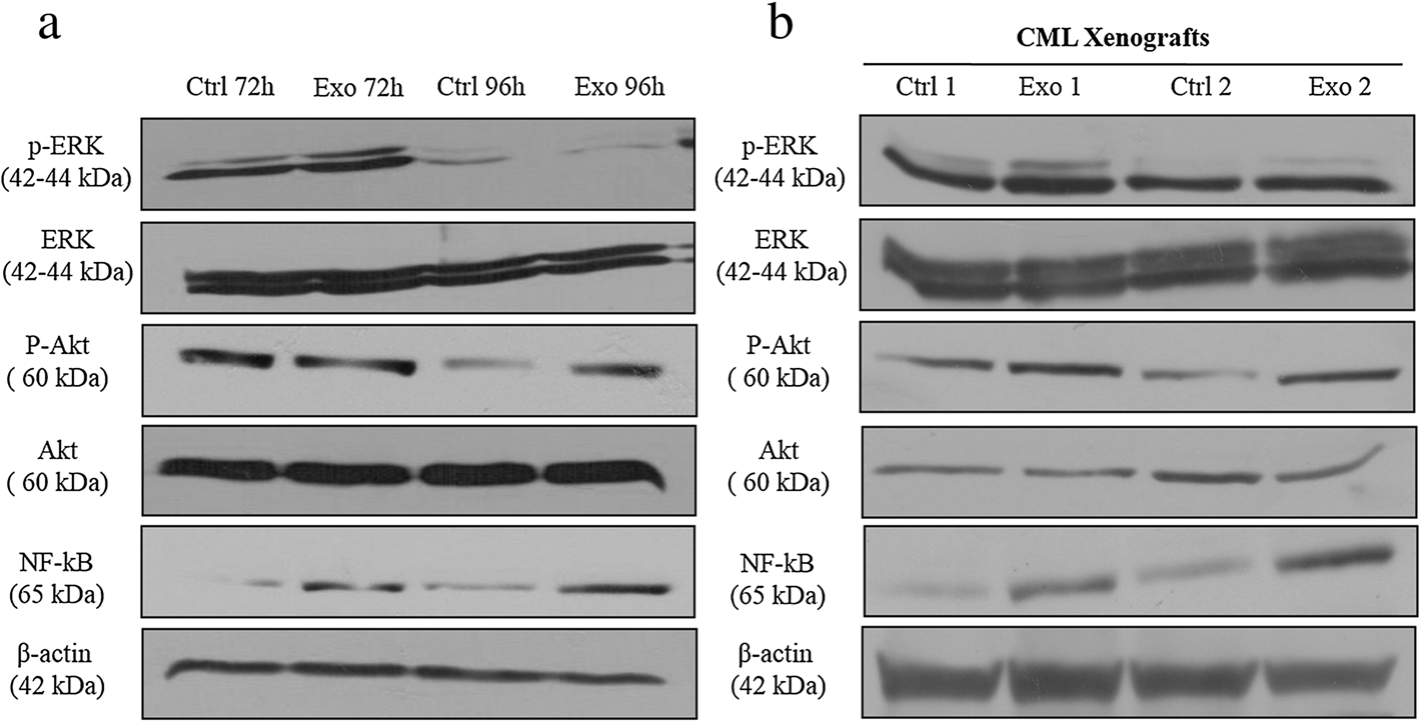
**CML Exosomes Trigger the** ***in Vitro*** **and** ***in Vivo*** **Activation of ERK/ Akt/ NF-Kb Pathways. (a)** Western blot analysis shows that treatment of LAMA84 with 10 μg/ml of LAMA84 exosomes for 72 or 96 hours triggers the phosphorylation of ERK and Akt without altering the total level of these proteins, and increases the expression of NF-kB. **(b)** A comparable effect can be observed in CML xenografts treated or not with exosomes.

To determine which signaling pathways are responsible for the activation of the pro-survival phenotype in exosome-treated LAMA84 cells, we performed Western blot analysis in the *in vitro* and *in vivo* samples. Since the PI3/Akt or MAPK/ERK pathways are responsible for the control of the growth and survival of cancer cells [18,19], we wondered whether exosomes are able to induce cancer cell proliferation through the activation of these signaling pathways. As shown in Figure 4a, 10 μg/ml of exosome treatment triggered a moderate phosphorylation of ERK after 72 hours of treatment. Moreover, after 96 hours of treatment, there was an increase in the phosphorylation of Akt. A significant increase in the phosphorylation of ERK or Akt could be observed in tumor biopsies from mice treated with exosomes compared with control mice (Figure 4b). Exosome treatment did not affect the total amount of these proteins. NF-kB is one of the key proteins that regulate cancer cell proliferation and survival by activating many pro-survival and anti-apoptotic genes, such as BCL-xl and survivin [20], as well as by interacting with other survival pathways, such as PI3/Akt [21]. Therefore we examined the level of this transcriptional factor. As shown in Figure 4, exosome treatment induced a significant increase in the level of NF-kB both *in vitro* and *in vivo*. These data suggest that CML exosomes stimulate the proliferation and survival of the producer cells *via* the activation of ERK, Akt and NF-kB.

### TGF-β1 is expressed in LAMA84-exosomes and modulates the SMAD pathway in CML cells

**Figure 5:**
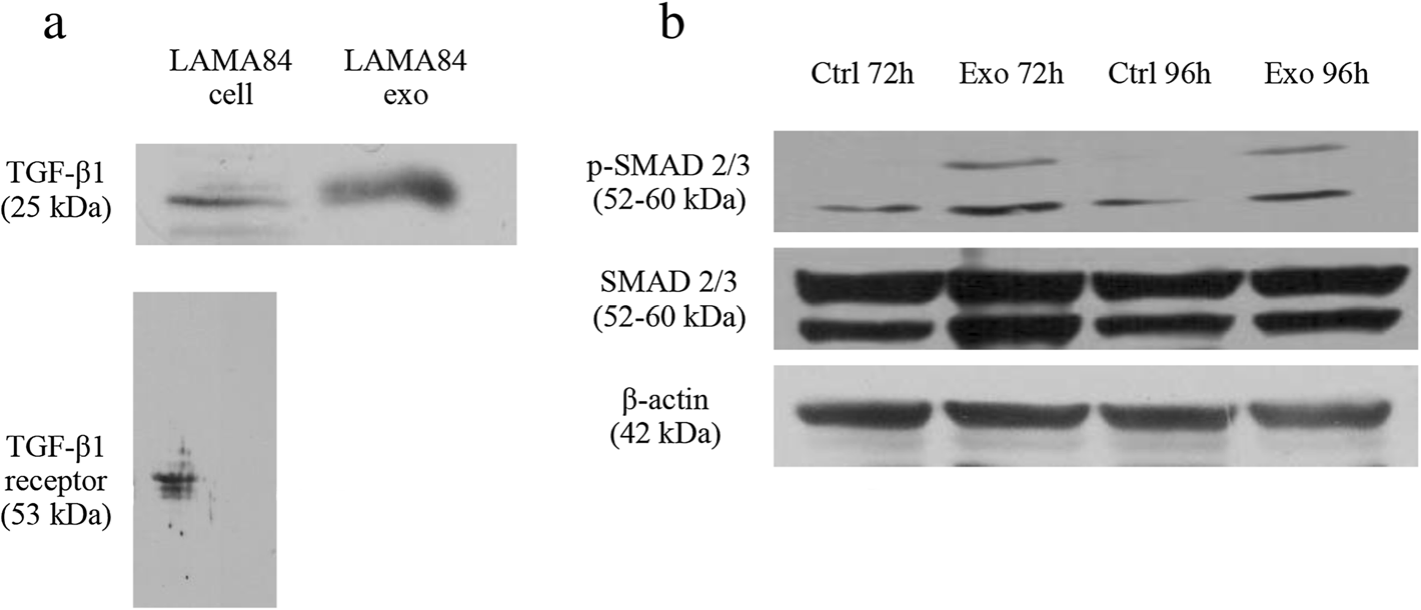
**TGF-β1 Is Expressed on CML Exosomes. (a)** Western blot analysis shows that LAMA84-derived exosomes express TGF- β1. Loading was based on equal protein amount (upper panel). LAMA84 cells express TGF- β1 receptor (lower panel). **(b)** After 72 or 96 hours of treatment of LAMA84 with 10 μg/ml of LAMA84-exosomes there is an increase in the phosphorylation of the downstream target of TGF-β1, SMAD 2/3; the treatment does not alter the total level of the protein.

In order to investigate which molecule is present in LAMA84 exosomes that could be responsible for the activation of pro-survival and proliferative pathways, we assessed by Western blot the expression of TGF-β1 in LAMA84-derived exosomes. As shown in Figure 5a (upper panel), TGF-β1 is enriched in exosomes compared with LAMA84 cells. Moreover, we observed that LAMA84 cells express the TGF-β1 receptor (Figure 5a, lower panel). It is well known that in cancer, the TGF-β1/TGF-β1 receptor complex controls various downstream signaling pathways, including Akt and MAPK signal transduction pathways, as well as the canonical SMAD dependent pathway [22].

As shown in Figure 5b, the treatment of LAMA84 with 10 μg/ml of CML exosomes for 72 or 96 hours induces the phosphorylation of SMAD 2/3 without altering the total protein level. These data suggest that CML-derived exosomes are able to establish an autocrine loop with their producer cells, possibly through a ligand-receptor interaction mediated by exosome-associated TGF-β1.

### Treatment of LAMA84 cells with TGF-β1 receptor inhibitor blocks exosome-mediated effects

**Figure 6:**
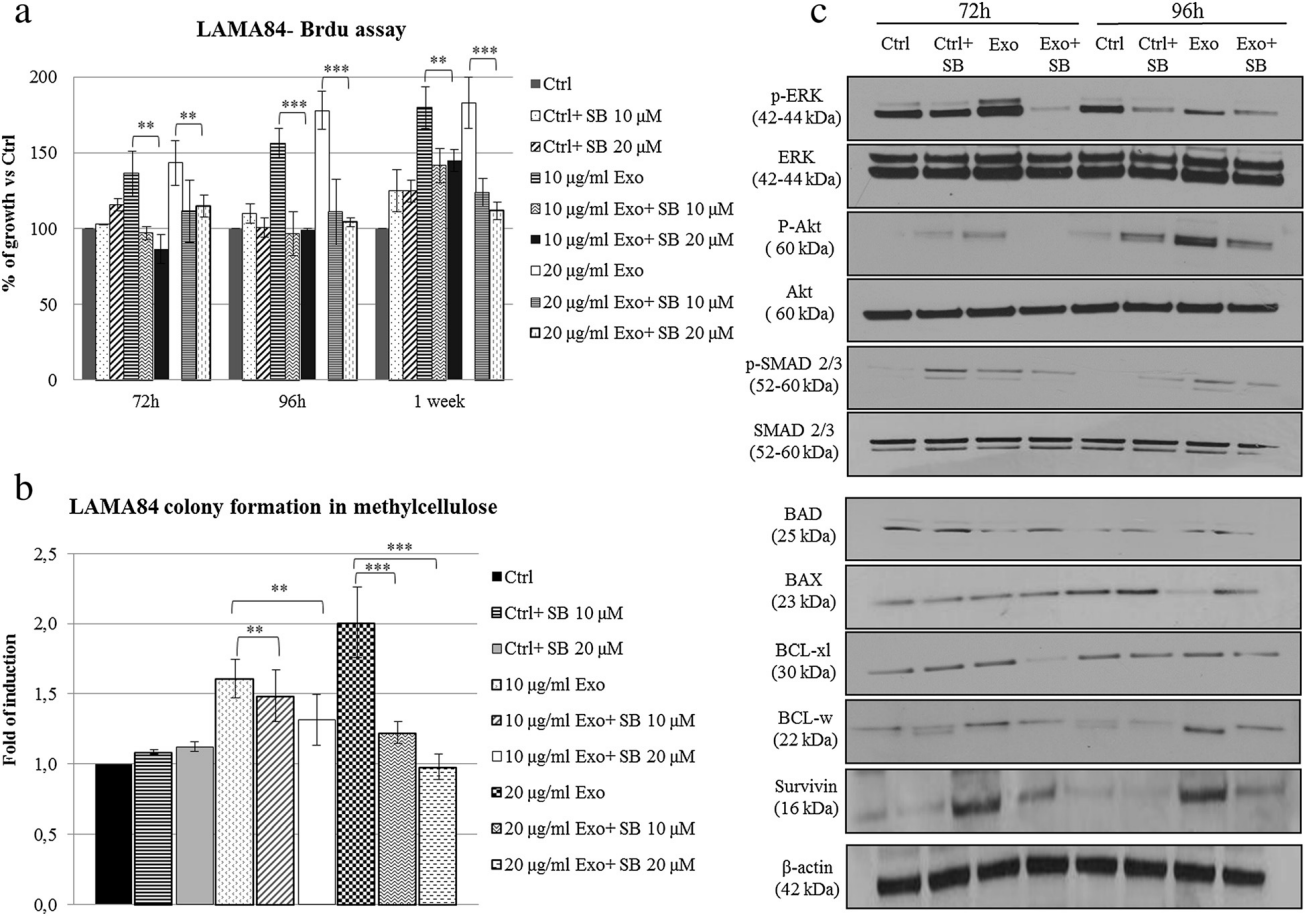
**Inhibition of TGF-β1 Receptor Signaling Reverses Exosome-Mediated Effects on CML Cells. (a)** Cell growth was evaluated by BrdU assay. LAMA84 cells were incubated for 72, 96 hours, or 1 week, with increasing doses of LAMA84-exosomes (10–20 μg/ml) plus 10 or 20 μM of SB. The values were plotted as a percentage of the control (untreated cells); each point represents the mean ± SD of three independent experiments. **(b)** Colony formation assay shows that the co-treatment of LAMA84 with 10 or 20 μg/ml of exosomes plus 10 or 20 μM of SB reverses exosome-mediated increase of LAMA84 colonies area (** p ≤ 0.01; ***p ≤ 0.001). **(c)** Western blot analysis shows that treatment of LAMA84 with 10 μg/ml of exosomes plus 10 μM of SB for 72 or 96 hours decreases the phosphorylation of ERK, Akt and SMAD 2/3 without altering total protein level. The co-treatment with SB reverses exosome-mediated decrease of the pro-apoptotic molecules (BAD, BAX) as well as the increase of the anti-apoptotic proteins (BCL-xl, BCL-w, survivin).

To further demonstrate the involvement of TGF-β1 signalling pathway, we co-treated CML cells with exosomes and TGF-β1 receptor inhibitor (SB). As shown in Figure 6a, the addition of 10 or 20 μM of SB significantly reduced the proliferation rate of exosome-treated cells and the size of exosome-stimulated colony areas without affecting the areas of control colonies (Figure 6b). Moreover the co-treatment with 10 μM of SB was able to significantly reduce exosome-stimulated increase of p-ERK and p-Akt without affecting the total protein levels. In addition, SB treatment reversed exosome-mediated activation of SMAD 2/3 (Figure 6c). In order to correlate the activation of TGF-β1 dependent pathways with the exosome-mediated activation of an anti-apoptotic phenotype in LAMA84 cells, we analyzed the levels of pro- and anti-apoptotic molecules in LAMA84 co-treated with exosomes and SB. As shown in Figure 6b the co-treatment with the inhibitor significantly reversed exosome-mediated decrease of BAD and BAX, as well as the increase of BCL-xl, BCL-w, and survivin. These data provide evidence of the involvement of TGF-β1 in exosome-mediated proliferation and survival of leukemic cells.

### Treatment of LAMA84 exosomes with TGF-β1 neutralizing antibodies inhibits LAMA84 proliferation and colony formation

**Figure 7:**
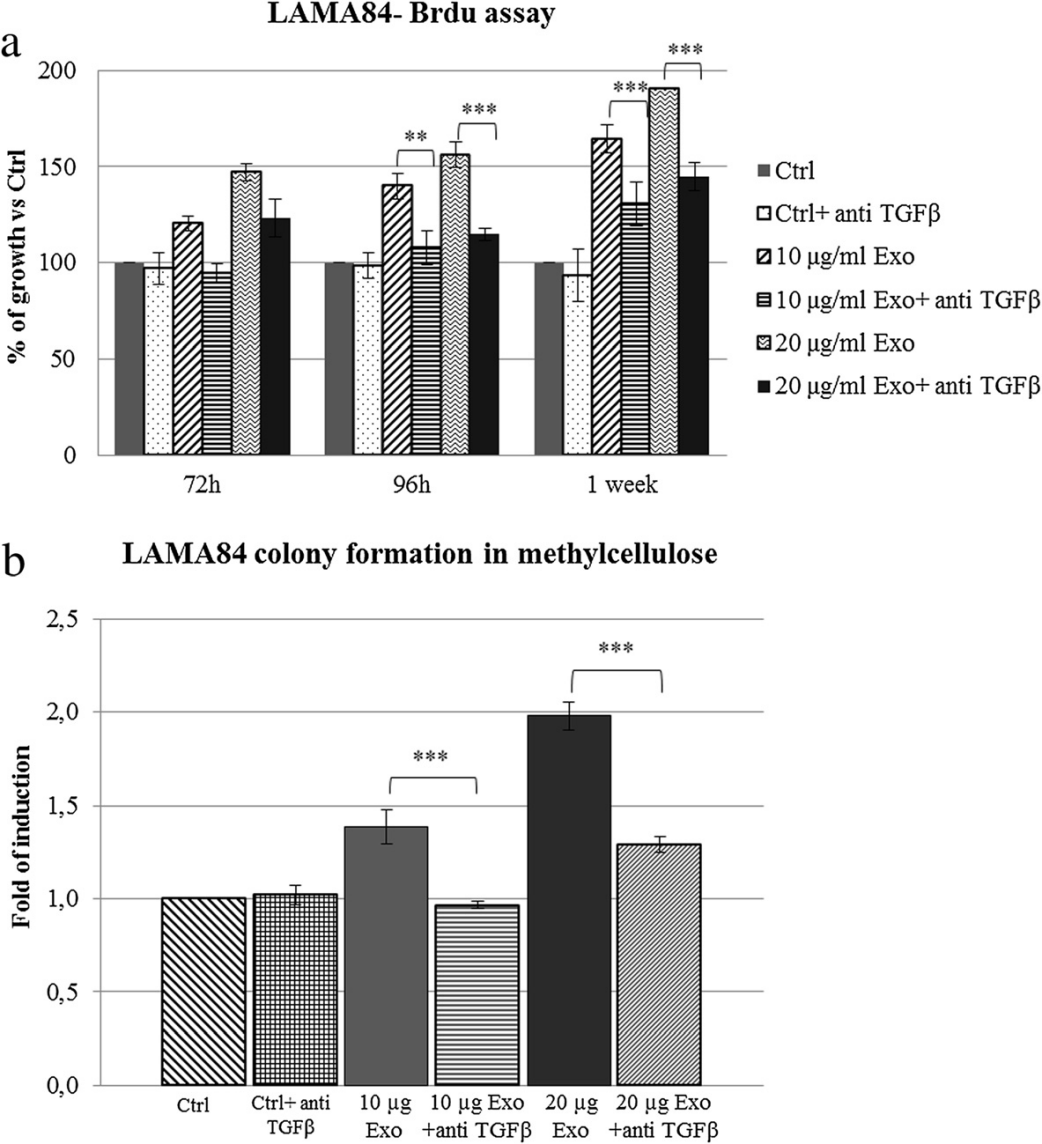
**Exosomal TGF- β1 inhibition reverses exosome- mediated increase of proliferation and colonies area of LAMA84 cells. (a)** Cell growth was evaluated by BrdU assay. LAMA84 cells were incubated for 72, 96 hours, or 1 week, with LAMA84-exosomes (10–20 μg/ml) pretreated or not with neutralizing anti TGF-β1 antibodies. The values were plotted as a percentage of the control (untreated cells); each point represents the mean ± SD of three independent experiments. **(b)** Colony formation assay shows that the co-treatment of LAMA84 with 10 or 20 μg/ml of exosomes pretreated with neutralizing anti TGF-β1 antibody reverses exosome-mediated increase of LAMA84 colonies area. Asterisks indicate a statistically significant values (** p ≤ 0.01; ***p ≤ 0.001).

In order to confirm the proliferative and pro-survival effects of exosomal TGF-β1 on producer cells, we first neutralized TGF β1 in LAMA84-derived exosomes, using an anti-TGF-β1 antibody, and we then treated LAMA84 cells with these vesicles. As shown in Figure 7 the stimulation of LAMA84 cells with TGF-β1 neutralizing antibody- treated exosomes significantly reverses CML cell proliferation induced by control exosomes (Figure 7a). Furthermore, pretreated exosomes doesn’t alter the size of LAMA84 colonies in methylcellulose (Figure 7b). Collectively, these results indicate that exosomal TGF-β1 interacts with TGF-β1 receptor on CML cells, leading to the increase of cell proliferation and survival.

## Discussion

We provide evidence that chronic myeloid leukemia-derived exosomes enhance tumor growth and survival through a direct autocrine effect. The autocrine stimulation is of crucial importance for the growth and survival of tumor cells, and may offer opportunities for treatment of patients by targeting growth factors and their receptors. Cancer cells are able to release nano-sized vesicles, called exosomes, which modulate tumor behavior in the surrounding microenvironment.

Exosomes are now considered an integral part of the intercellular microenvironment, and may act as regulators of cell-to-cell communication [5]. Increasing evidence shows that these vesicles are able to induce phenotypic changes in neighboring cells by activating specific cell signaling pathways that lead to cancer progression. We recently reported evidence of the paracrine mechanism by which chronic myeloid leukemia-derived exosomes promote cancer cell survival by inducing phenotypic changes in endothelial and stromal cells [8,9]. Exosomes can also act in an autocrine manner, acting on their producer cells. Koga and colleagues have shown that breast-cancer-derived exosomes stimulated the proliferation of the parental cells [23]. A few years later, Qu and colleagues reported that gastric cancer exosomes promoted tumor cell proliferation, at least in part, by activation of PI3K/Akt and mitogen-activated protein kinase/extracellular-regulated protein kinase pathways [10]. Here, we show that the promotion of proliferation and colony formation of CML cell lines by CML-exosomes points to an important role of autocrine mechanisms mediated by these vesicles in the biology of CML. The proliferative effect of exosome stimulation was also confirmed in our *in vivo* CML xenograft model, supporting the *in vitro* evidence. The extracellular signal-regulated kinase (ERK) signaling pathway is a major determinant in the control of diverse cellular processes, such as proliferation and survival, and together with the PI3K/Akt pathway is often up-regulated in many cancers. Emerging evidence has shown that tumor-derived exosomes are able to remodel the tumor microenvironment by regulating these signaling pathways in target cells. Al-Nedawi and colleagues reported that exosome-derived oncogenic EGFR from human squamous cell carcinoma activated, in endothelial cells, MAPK and Akt cell signaling pathways, promoting VEGF expression [24]. Furthermore, various studies of pancreatic- and colorectal-derived exosomes have indicated a transfer of growth factor encoding mRNA (VEGF, HGF, IL-8, CD44H) to tumor-associated monocytes, leading to an anti-apoptotic response due to the activation of the Akt pathway [25].

In our study, we correlate the *in vitro* and *in vivo* exosome-mediated stimulation of CML growth and survival with a deregulated activation of both ERK and Akt pathways. In particular, Western blot analysis showed an increase in the phosphorylation of both kinases after exosome treatment. Our data on CML exosomes confirm what Qu and collaborators previously described in exosome-treated gastric cancer cell lines [10]. In addition to the activation of ERK and Akt pathways, we found in exosome-treated cells an up-regulation of the anti-apoptotic NF-kB signal transduction pathway, a downstream target of Akt. It is well known that Akt promotes cells survival through the activation of NF-kB together with the down-regulation of pro-apoptotic molecules and the up-regulation of pro-survival molecules [26-28]. In our *in vitro* and *in vivo* models, we found an increase in the mRNA and protein levels of the pro-apoptotic molecules BAD, BAX and PUMA, together with the increase of the pro-survival molecules BCL-XL, BLC-w, and survivin. Our data on the regulation of pro- and anti-apoptotic molecules, together with the activation of ERK and Akt pathways, suggest that CML exosomes trigger an anti-apoptotic phenotype in the producer cells. Therefore, the release of exosomes by CML cells could contribute to the progression of leukemia through the triggering of an autocrine loop, thus representing a possible target for new therapies.

Among the molecules that act on cancer cells through the establishment of an autocrine loop, TFG- β1 is a crucial cytokine for CML progression. The transforming growth factor 1 (TGF-β1) is a multifunctional cytokine depending on cellular context. It is known for its cytostatic effects in premalignant states and its pro-oncogenic activity in advanced cancers. In additional to the canonical SMAD pathway, TFG- β1 is able to regulate the so-called non-SMAD signaling pathways, which include PI3K/Akt and MAPKs, two key regulatory pathways of cancer cell proliferation and survival [22]. Various studies have underlined the role of TGF-β1 in leukemia progression. Naka and colleagues found that the TGF- β1 pathway is critical in the maintenance of leukemia-initiating cells by controlling FOXO localization. Furthermore, studies have shown that the combination of TGF-β inhibitors with imatinib efficiently depleted leukemia-initiating cells and retarded CML development [15]. Furthermore, Zhu and colleagues have shown that the BCR-ABL oncogene up-regulated TGF-β1 expression, which in turn activated PI3K/Akt/NF-kB/MMP9 signaling pathways. The TGF-β1-mediated signaling led to an enhanced recruitment and mobilization of leukemia stem cells to the peripheral circulation [13]. Here we add another piece of information on the role of TGF-β1 in CML progression.

We found that TGF-β1 is enriched in LAMA84-derived exosomes. Previous studies have reported the presence of TGF-β1 in both cancer and non-cancer cells, underscoring its role on exosome-stimulated effects on recipient cells [16,29,30]. To further determine the autocrine involvement of exosomal-TGF-β1 in the stimulation in leukemic cells, we used a commercially available TGF-β1 receptor inhibitor or a specific neutralizing antibody to exosomal TGF-β1. Our study showed that the exosome- stimulated increase of cell proliferation, and the activation of an anti-apoptotic phenotype can be inhibited by blocking TGF-β1 mediated pathways. Some studies have reported that TGF-β1 levels are often elevated in human tumors [31,32]. As a result of the wide variety of effects of TGF-β1 on tumorigenesis, blocking the TGF-β pathway may provide multiple therapeutic opportunities. There are many TGF-β1 signaling antagonist agents under development at both the pre-clinical and clinical stages [33]. Naka and colleagues demonstrated that drug resistance. in CML stem cells is due to TGF-β1 in the microenvironment, and that the treatment of human CML stem cells with a TGF-β1 inhibitor inhibited their clonogenic activity *in vitro* [15]. Therefore, it is conceivable that the anti-apoptotic effects of CML-derived exosomes on the producer cells may be partially due to the presence of TGF-β1 on CML exosomes. Our data underline the importance of evaluating the role of leukemia-derived exosomes for the development of combination therapies that potentiate the effects of imatinib treatment.

## Conclusions

**Figure 8:**
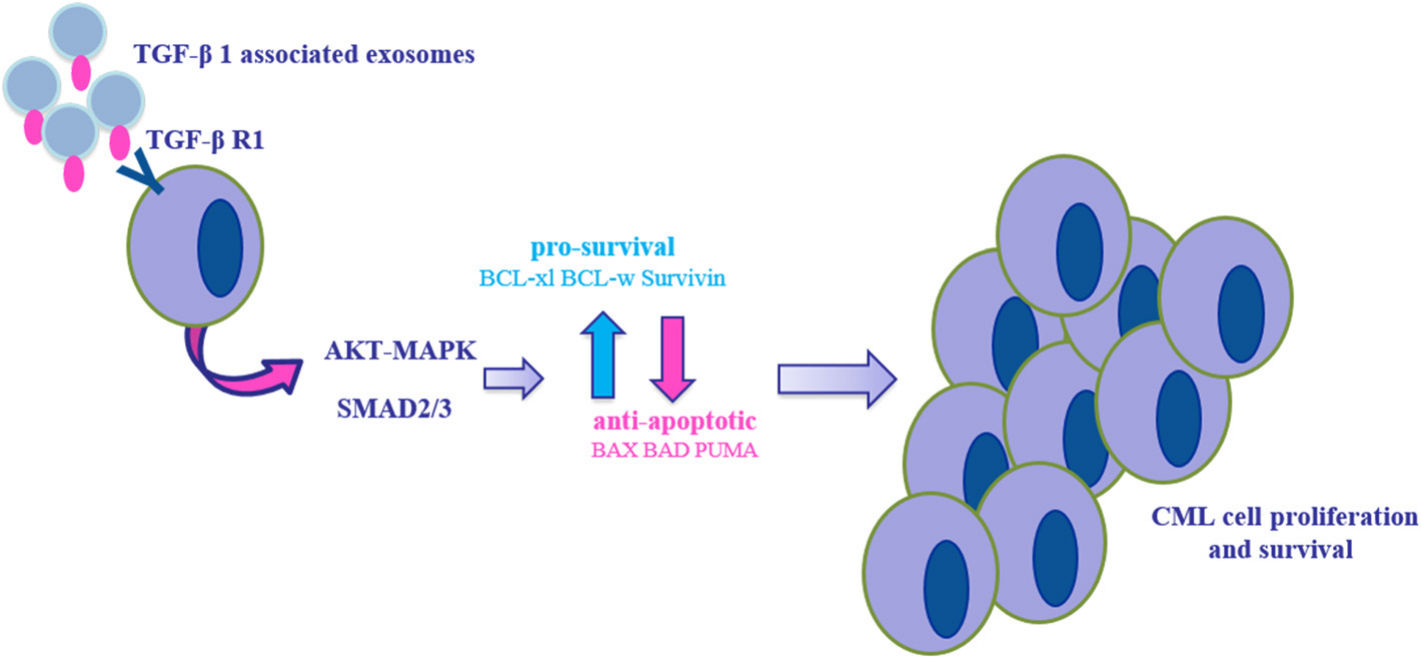
**Working Hypothesis on Exosome-mediated Autocrine Loop.** CML derived exosomes promote proliferation and survival of leukemic cells through the establishment of an autocrine signaling loop mediated by exosome-associated TGF-β1.

In summary, we have identified an autocrine role for CML-derived exosomes that lead to the activation of leukemia growth and survival (Figure 8). These data provide a new understanding of the role of exosomes in cancer biology. The extensive autocrine stimulation that occurs in tumors has significant implications for innovative therapeutic and biomarker strategies for leukemia.

## Methods

### Ethics statement

All animal experiments were conducted in full compliance with University of Palermo and Italian Legislation for Animal Care. The Dipartimento di Biopatologia e Biotecnologie Mediche e Forensi (DiBiMef) Review Board approved this study.

### Cell culture and reagents

The human chronic myeloid leukemia cell line, LAMA84, was obtained by DSMZ (Braunschweig, Germany) and cultured in RPMI 1640 medium (Euroclone, UK), supplemented with 10% fetal bovine serum, 2 mM L-glutamine, 100 U/ml penicillin, and 100 μg/ml streptomycin (Euroclone, UK). SB431542 (Cayman Chemical, Michigan, USA) was solubilized at 10 mM stock solution in DMSO, and stored at -20°C. Working dilutions were prepared in medium. All other reagents were purchased from Sigma-Aldrich (St. Louis, MO, USA), if not cited otherwise.

### Exosome preparation

Exosomes released by LAMA84 CML cells after a 24-hour culture period in presence of FBS previously ultracentrifuged (vesicle free media) were isolated from conditioned culture medium by differential centrifugation, as previously described [6,8]. Briefly, culture medium was centrifuged subsequently for 5 min at 300 × g, 15 min at 3,000 × g, 30 minutes at 10,000 × g and ultracentrifuged 90 min at 100,000 × g in a Type 70 Ti, fixed angle rotor. Peletted exosomes were washed and then resuspended in PBS. To further verify the identity of vesicles as exosomes, we isolated exosomes on a 30% sucrose/D2O cushion. Vesicles contained in the cushion were recovered, washed several times, ultracentrifuged for 90 min in PBS and collected for use. Exosome protein content was determined with the Bradford assay (Pierce, Rockford, IL, USA). On average, we recovered 10 micrograms of vesicles from 3×10^6^ cells; this amount of exosomes was then used to treat 1×10^6^ cells.

### Proliferation assay (BrdU assay)

Cell proliferation was assessed with a BrdU labeling ELISA kit (Calbiochem, San Diego, CA, USA) according to the manufacturer’s protocol. Briefly, LAMA84 cells were seeded at a density of 0.1×10^6^ in a 96-well plate and exposed to escalating doses of LAMA84-exosomes (5–10–20 μg/ml), pretreated or not with anti- TGF-β1 antibody, and in the presence or not of SB431542 (10, 20 μM) for up to one week. Twenty microliters of BrdU were added to the cells 24 hours before each time point. The absorbance was measured at dual wavelength (450 nm and 540 nm). Means and standard deviations generated from three independent experiments are reported as the percentage of growth versus control (untreated cells). Cell proliferation curves were derived from these data with Microsoft Excel software.

### Colony formation assay

LAMA84 cells were plated in 6-well (2000 cells/ml/well) in Iscove’s-methylcellulose medium (Methocult H4230, Stem Cell Technologies, Vancouver, Canada) containing or not LAMA-derived exosomes (1, 5, 10, 20, 50 μg/ml), pretreated or not with anti- TGF-β1 antibodies, and in the presence or not of SB431542 (10, 20 μM). After 14 days of culture, LAMA84 colonies were observed by phase-contrast microscopy and photographed. The area of twenty LAMA84 colonies per condition was measured with the IMAGE-J software (http://rsbweb.nih.gov/ij/).

### CML mouse xenograft

Male NOD/SCID mice, four-to-five weeks old, were purchased from Charles River (Charles River Laboratories International, Inc, MA, USA) and acclimated for a week prior to experimentation. Mice received filtered water and sterilized diet *ad libitum*. Animals were observed daily and clinical signs were noted. Mice were randomly assigned to two groups of seven. Each mouse was inoculated subcutaneously in the right flank with viable single human LAMA84 cells (2 × 10^7^) suspended in 0.2 ml of PBS with: (i) PBS (vehicle) or (ii) LAMA84-derived exosomes (100 μg/mouse). The day of injection was considered Day 0. Treatments with PBS (control group) or exosomes were repeated twice a week, in the tumor site. Xenograft tumors were measured and mice were weighed twice a week. Tumor volume was determined by calliper with the following formula: LxW^2^/2 = mm^3^ where L and W are the longest and shortest perpendicular measurements in millimeters, respectively. The same formula was used to calculate tumor weights assuming that 1 mm^3^ = 1 mg. Animals were euthanized at day 50, the tumor resected, and the tumor weights measured. Xenografts were resuspended in RNA later for further RNA isolation in 4% formaldehyde for immunohistochemical analysis or maintained dried at -80°C for protein isolation.

### RNA extraction and real-time PCR

LAMA84 were grown in 12-well plates and treated with 5 or 10 μg/ml of LAMA84- exosomes for 72, 96 hours, or 1 week. Tumor biopsies soon after removal were stored in RNAlater solution (Applied Biosystems, Foster City, California, USA). Each sample was lysed in a tissue homogenizer. RNA was extracted using the commercially available Illustra RNAspin Mini Isolation Kit (GE Healthcare, Little Chalfont, Buckinghamshire, UK), according to manufacturer’s instructions. Total RNA was reverse-transcribed to cDNA using the High Capacity cDNA Reverse Transcription Kit (Applied Biosystem). RT-QPCR was performed in 48-well plates using the Step-One Real-Time PCR system (Applied Biosystem).

For quantitative SYBER®Green realtime PCR, reactions were carried out in a total volume of 20 μl containing 2× SYBR®Green I Master Mix (Applied Biosystems), 2 μl cDNA and 300 nM forward and reverse primers. Primers sequence were:

GAPDH (5′ATGGGGAAGGTGAAGGTCG3′, 5′GGGTCATTGATGGCAACAATAT3′),.

BAD (5′CCGAGGAGCAGGAAGACTC′3, 5′GGTAGGAGCTGTGGCGACT′3),.

BAX (5′CCTGTGCACCAAGGTGCCGGAACT3′, 5′CCACCCTGGTCTTGGATCCAGCCC3′),.

BCL-w (5′AGTTCGAGACCCGCTTCC′3, 5′CCCGTCCCCGTATAGAGC′3),.

BCL-xl (5′CTGAATCGGAGATGGAGACC′3, 5′TGGGATGTCAGGTCACTGAA′3),.

PUMA (5′GGAGCAGCACCTGGAGTC′3, 5′TACTGTGCGTTGAGGTCGTC′3),.

Survivin (5′CTCAAGGACCACCGCATCTC′3, 5′CAGCCTTCCAGCTCCTTGAA′3),.

All obtained from Invitrogen (Foster City, CA, USA). Real-time PCR was performed in duplicates for each data point. Relative changes in gene expression between control and treated samples were determined with the ΔΔCt method. Levels of the target transcript were normalized to a GAPDH endogenous control, constantly expressed in all samples (ΔCt). For ΔΔCt values, additional subtractions were made between treated samples and control ΔCt values. Final values were expressed as fold of induction.

### Western blot

LAMA84 cells were treated or not for 72 or 96 hours with 10 μg/ml of LAMA84-derived exosomes in the presence or not of 10 μM SB431542.Total protein cell lysates or exosome lysates were obtained and analyzed by SDS-PAGE in reducing- or non-reducing conditions followed by Western blotting, as previously described [6]. Antibodies used in the experiments were anti-Akt, phospho-Akt, Erk 1/2, phospho Erk 1/2, NF-kB, SMAD 2/3, phospho SMAD 2/3, β-actin, Alix (all from Cell Signalling Technology, MA, USA), anti-BAD, BAX, BCL-xL, BCL-w, survivin, TGF-β1, TGF- β1 receptor, Tsg101, CD81, CD63, Hsc70 and Calnexin (all from Santa Cruz Biotechnology, CA, USA).

### Immunohistochemical staining

Mice xenografts were immediately fixed with 4% formaldehyde and embedded in paraffin. IHC for BAX and BCL-w was performed on 5-μm-thick paraffin-embedded sections from tumor biopsies from mice treated with exosomes or with PBS (control). Isotype-matched irrelevant antibodies were used as a negative control. Following rehydration, the antigen was unmasked for 45 minutes in a 95°C microwave with Dako Target Retrieval Solution (pH 6; Dako, Carpinteria, California, USA). Endogenous peroxidase was blocked for 10 minutes with Dako peroxidase blocking reagent, and non-specific binding was blocked for 20 minutes with Dako protein block. The primary antibodies anti-human BAX and BCL-w (1:100 diluition, Santa Cruz Biotechnology) were added and incubated for 1 hour at room temperature. For control staining, primary antibodies were replaced with irrelevant isotype-matched antibodies (AbCam, Cambridge, UK). The slides were then incubated for 30 minutes with peroxidise-conjugated Dako EnVision polymer, and peroxidase activity was visualized with diaminobenzidine chromogen (Dako). Slides were lightly counterstained with hematoxylin before dehydration and mounting in DePex (VWR International, Oslo, Norway).

### TGF-β neutralization

Exosomes were incubated with TGF-β1 neutralizing antibody (20 mg/mL, Abcam, Cambridge, UK) for 2 hours at 37°C. LAMA84 cells were treated as indicated in results and figure legends.

### Statistical analysis

Data are expressed as means ± SD of three independent experiments. Statistical analysis was done with a paired sample *t*-test. Differences were considered significant when p ≤ 0.05.

## Additional file

Additional file 1: Figure S1. LAMA84 cells-Derived Exosomes characterization. (a) Ponceau staining of LAMA84 cell lysates and exosomes. (b) Detection of Calnexin, CD81, Alix, Tsg10, Hsc 70 and CD63 in 30 μg of cell and exosomes lysates. Exosomes are positive for CD81, Alix, Tsg101, Hsc70 and CD63 but negative for the endoplasmic reticulum protein, Calnexin.

## Abbreviations

CML: Chronic myeloid leukemia
ERK1/2: Extracellular signal-regulated kinase1/2
MAPK: Mitogen-activated protein kinase
MVB: Multivesicular body
PI3K/Akt: Phosphatidylinositol 3' –kinase/ Akt
TGF-β1: Transforming Growth Factor β1
