# Supplementary figures and images for: Chronic myeloid leukemia-derived exosomes promote tumor growth through an autocrine mechanism
Source: Cell Commun Signal. 2015 Feb 3;13:8. doi: 10.1186/s12964-015-0086-x (PMC4320527; doi:10.1186/s12964-015-0086-x)

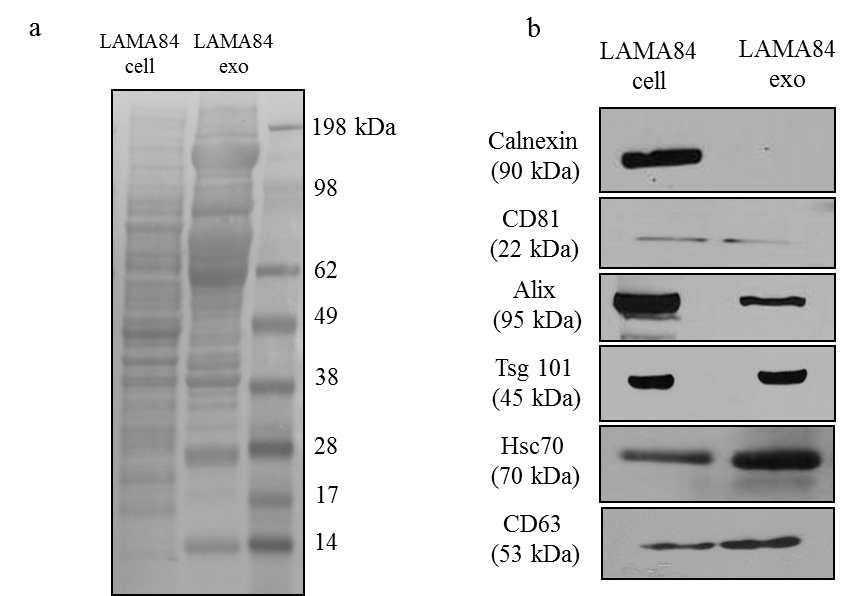

Supplement: Additional file 1: Figure S1. — LAMA84 cells-Derived Exosomes characterization. (a) Ponceau staining of LAMA84 cell lysates and exosomes. (b) Detection of Calnexin, CD81, Alix, Tsg10, Hsc 70 and CD63 in 30 μg of cell and exosomes lysates. Exosomes are positive for CD81, Alix, Tsg101, Hsc70 and CD63 but negative for the endoplasmic reticulum protein, Calnexin. [file 12964_2015_86_MOESM1_ESM.tiff]
